# Cutaneous Squamous Cell Carcinoma Across the Pre-COVID-19, COVID-19 and Post-COVID-19 Eras: Epidemiology, Risk Stratification, Tumour Aggressiveness, and Clinical Outcomes

**DOI:** 10.3390/dermatopathology13030036

**Published:** 2026-08-05

**Authors:** Martin Manole, Iuliu Gabriel Cocuz, Alexandru-Constantin Ioniță, Maria Baldea, Carla-Antonia Peterdeak, Adrian Horațiu Sabău, Maria-Cătălina Popelea, Emőke Andrea Szász, Andreea Raluca Cozac-Szőke, Andreea Cătălina Tinca, Diana Maria Chiorean, Ovidiu Simion Cotoi

**Affiliations:** 1 Faculty of Medicine, “George Emil Palade” University of Medicine, Pharmacy, Sciences and Technology of Targu Mures, 540142 Targu Mures, Romania; martinmanolemd98@gmail.com (M.M.); alexionita2@gmail.com (A.-C.I.); maria.baldea@yahoo.com (M.B.); karlapeterdeak@yahoo.com (C.-A.P.); adrian-horatiu.sabau@umfst.ro (A.H.S.); popelea.maria@gmail.com (M.-C.P.); emoke.szasz@umfst.ro (E.A.S.); andreea-raluca.szoke@umfst.ro (A.R.C.-S.); andreea-catalina.tinca@umfst.ro (A.C.T.); diana.chiorean@umfst.ro (D.M.C.); ovidiu.cotoi@umfst.ro (O.S.C.); 2 Clinical Pathology Department, Mures Clinical County Hospital, 540011 Targu Mures, Romania; 3 Pathophysiology Department, “George Emil Palade” University of Medicine, Pharmacy, Sciences and Technology of Targu Mures, 540142 Targu Mures, Romania; 4 Histology Department, “George Emil Palade” University of Medicine, Pharmacy, Sciences and Technology of Targu Mures, 540142 Targu Mures, Romania

**Keywords:** squamous cell carcinoma, NMSC, COVID-19, epidemiology, histopathology

## Abstract

**Background/Objectives**: Cutaneous squamous cell carcinoma (cSCC) is the second most common non-melanoma skin cancer (NMSC) and represents the leading cause of NMSC-related deaths. Despite its growing global burden, comprehensive epidemiological and clinicopathological data from Easter Europe remains limited. This study aimed to evaluate the epidemiological, clinical, histopathological, and surgical characteristics of cSCC diagnosed before, during and after the COVID-19 pandemic. **Methods**: We conducted a retrospective, descriptive observational study including 332 lesions diagnosed between January 2017 and December 2025 at the Clinical Pathology Department of the Mureș Clinical County Hospital. Demographic, epidemiological, topographic, histopathologic, surgical, and volumetric parameters were analyzed. Tumours were stratified into low-, high-, and very-high-risk categories according to the National Comprehensive Cancer Network (NCCN) criteria. **Results**: The cohort demonstrated a significant male predominance (*n* = 193 vs. *n* = 139; *p* = 0.0355), with females presenting a higher median age (77 vs. 75; *p* = 0.0489). Lesions were predominantly located in the head and neck region (*n* = 216; *p* < 0.0001), which was significantly associated with very-high-risk tumours (*p* = 0.0051). Low-risk tumours accounted for 62.35% of cases, while high-risk and very-high-risk lesions comprised 19.88% and 17.77%, respectively (*p* < 0.0001). Ulcerations were strongly associated with very-high-risk tumours (*p* < 0.0001). Poor differentiation was more frequent outside the head and neck region (*p* < 0.0001) and varied significantly across the pandemic periods (*p* = 0.0349). Tumoral and excision volumes were higher in very-high-risk (*p* = 0.0070; *p* = 0.0004) and ulcerated tumours (*p* < 0.001), with a peak in volume during the COVID-19 period (*p* < 0.0001). A decrease through the years of diagnosis was observed in tumoral volumes (*r* = −0.2295; *p* < 0.0001) and patients showed a weak positive correlation with diagnosis year (*r* = +0.13; *p* = 0.019). **Conclusions**: The study provides an epidemiological and clinicopathological characterization of cSCC within one of the largest Romanian cohorts to date. Tumour aggressiveness was primarily driven by histopathological and topographical features rather than demographic factors. While the COVID-19 pandemic did not induce persistent changes in tumour risk profiles or surgical outcomes, it influenced diagnosis timing and tumour burden. These findings highlight the importance of incorporating temporal and emerging systemic factors, such as pandemics, and infectious events, into future epidemiological models to improve preparedness, early detection, future treatment schemes, and risk stratification in cSCC.

## Simple Summary

Cutaneous squamous cell carcinoma (cSCC) is responsible for most deaths related to non-melanoma skin cancer (NMSC). Understanding which tumours are more aggressive is essential for selecting the most appropriate treatment and improving patient outcomes. In this study, we analyzed one of the largest Romanian cohorts of patients with cSCC, including cases diagnosed before, during and after the COVID-19 pandemic. We found that tumour-related features, particularly their anatomical location, microscopic characteristics, ulceration, and volume/size, were more important indicators of aggressiveness than patient characteristics such as age or sex. Our findings provide valuable epidemiological and clinicopathological data from an Eastern European population, where evidence remains limited. These results may support earlier identification of high-risk tumours, improve clinical risk stratification and surgical decision-making, and serve as a foundation for future dermatology, dermatopathology and population-based research on cSCC.

## 1. Introduction

Non-melanoma skin cancers (NMSCs) include predominantly basal cell carcinoma (BCC) and cutaneous squamous cell carcinoma (cSCC) [1,2]. Although BCC accounts for the majority of NMSC cases, it is characterized by exceptionally low metastatic potential, resulting in an almost negligible mortality rate [1,2]. In contrast, cSCC, while less common, exhibits substantially greater local aggressiveness and metastatic potential, making it one of the leading causes of NMSC-related deaths [3]. According to the World Health Organisation (WHO), approximately 1.23 million new NMSC cases and 64,400 related deaths occurred worldwide in 2022 [2,4,5]. By 2050, these figures are projected to rise to approximately 2.70 million cases and more than 150,000 deaths, highlighting the rapidly increasing global burden of NMSC [2,4,5].

cSCC originates from epidermal keratinocytes, specifically those of the spinous layer of the epidermis [6]. Risk factors include certain precursor lesions such as actinic keratosis (AK) [7] and Bowen’s disease (BD) [8,9]. Male gender, advanced age, and Fitzpatrick type I and II skin phenotypes are also associated with an increased risk [8,9]. Genetic predisposition and a positive family history play an important role, particularly in the context of inherited disorders such as *xeroderma pigmentosum*, *albinism*, *epidermodysplasia verruciformis*, and *Muir–Torre syndrome* [8,9]. In addition, exposure to certain chemical compounds, ionizing radiation, immunosuppressive medication and chronic inflammatory conditions may further increase the risk of developing cSCC [8]. However, the most significant risk factor remains chronic exposure to ultraviolet (UV) radiation, which ultimately induces mutations in genes such as *TP53*, *NOTCH1*, *NOTCH2*, *CDKN2A*, and *NRAS* [7,8,9].

The pathophysiology of cSCC is driven by cumulative genetic and epigenetic alterations. The UV-induced mutations, particularly *p53* inactivation [10,11], disrupt cell-cycle regulation and apoptosis, while alterations in *CDKN2A*, *TERT*, *NOTCH1/NOTCH2*, *TP63* and *RIPK4* promote impaired keratinocyte differentiation, prolonged cellular survival, uncontrolled proliferation, angiogenesis, and aggressive tumour behaviour [9,10,11,12,13]. Collectively, these molecular alterations create a tumour microenvironment that favours malignant progression, invasion, and metastasis [13]. Epigenetic alterations, including hypermethylation and aberrant histone modifications, further promote genomic instability, tumour progression, and invasion [9,14,15,16].

From a histopathological perspective, cSCC presents multiple subtypes, with distinct morphological features and biological behaviour [17]. These subtypes are stratified into risk categories according to their aggressiveness, guiding treatment and prognosis [18].

Disease progression commonly occurs through BD or cSCC in situ, characterized by full-thickness epidermal atypia with an intact basement membrane [17,18,19]. The acantholytic (adenoid or pseudoglandular) subtype is a rare, aggressive variant characterized by loss of cohesion between atypical keratinocytes, forming pseudoglandular or vascular-like spaces [20,21,22]. The clear cell subtype contains tumour cells with abundant clear or pale cytoplasm and should be distinguished from clear cell acanthoma and metastatic clear cell renal carcinoma [18]. The sarcomatoid subtype of cSCC consists of malignant spindle cells and often requires immunohistochemical (IHC) confirmation using *p40* or *p63* [23]. Together with the adenosquamous subtype, it is classified by the NCCN (National Comprehensive Cancer Network) as very high risk because of its increased likelihood of recurrence, metastasis, and disease-specific mortality [18]. Desmoplastic cSCC is characterized by a densely collagenized stroma, frequent perineural invasion and positivity for *p63* while remaining negative for SOX10 and S100 [18]. In contrast, keratoacanthoma and verrucous carcinoma generally exhibit a more indolent course with minimal metastatic potential [18,24,25,26].

In the pathogenesis of cSCC, an emerging area of research requiring further investigation is the potential role of oncogenic viral agents, such as human papillomavirus (HPV), which has been shown to induce immunosuppression during the early stages of infection. Viral proteins may mediate the degradation of tumour suppressors such as *p53* and *pRb*, thereby facilitating accelerated clonal proliferation of keratinocytes [27,28]. Similar mechanisms have been suggested in the context of SARS-CoV-2 infection, a virus associated with the induction of T-cell exhaustion, the triggering of cytokine storms, and the establishment of a prolonged inflammatory state, with possible long-term immunosuppressive consequences [29,30].

**Figure 1:**
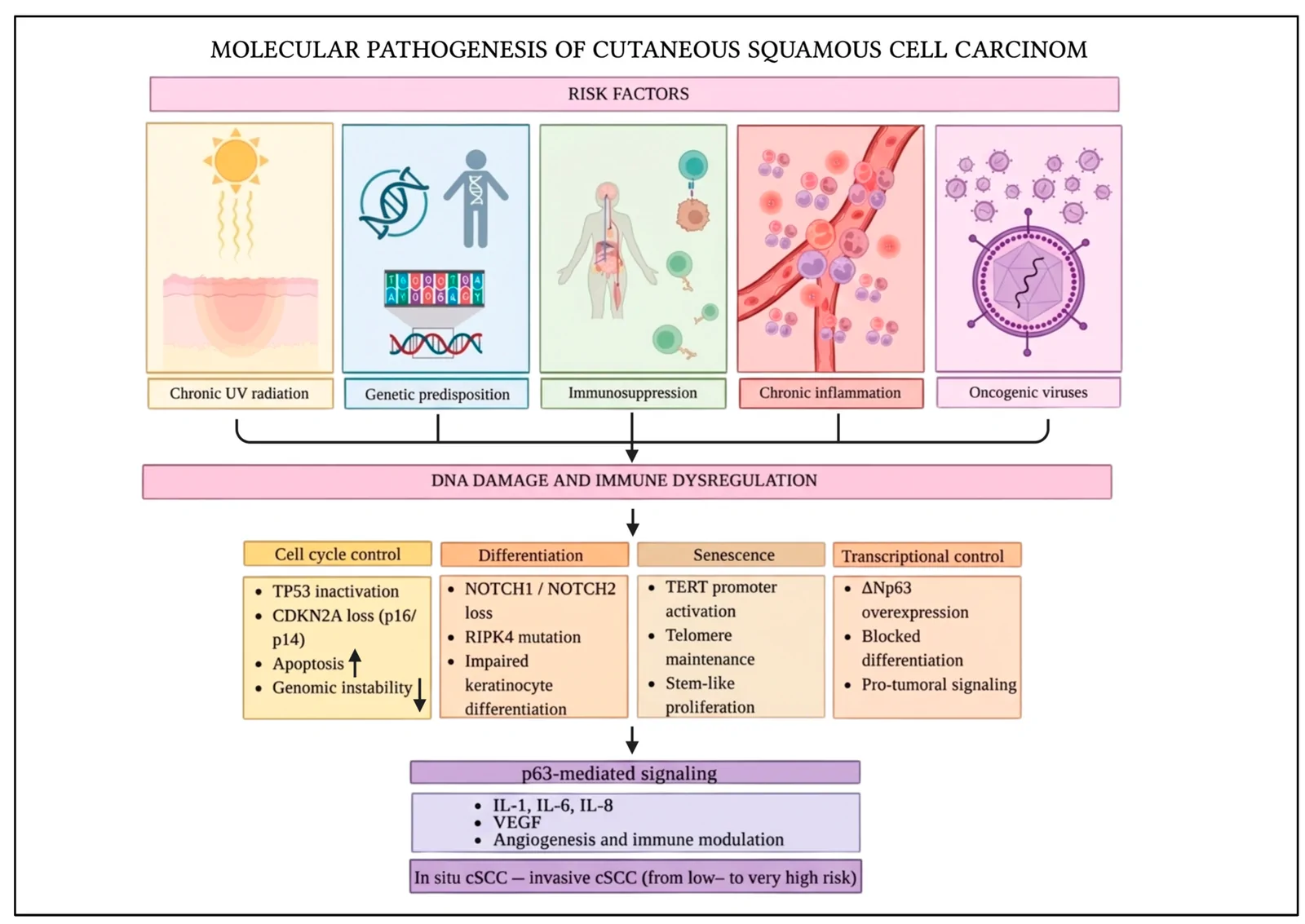
Molecular pathogenesis of cutaneous squamous cell carcinoma (created in BioRender; Manole, M. (2026) https://BioRender.com/6tvdil7) (URL accessed on 26 June 2026) [33].

**Figure 2:**
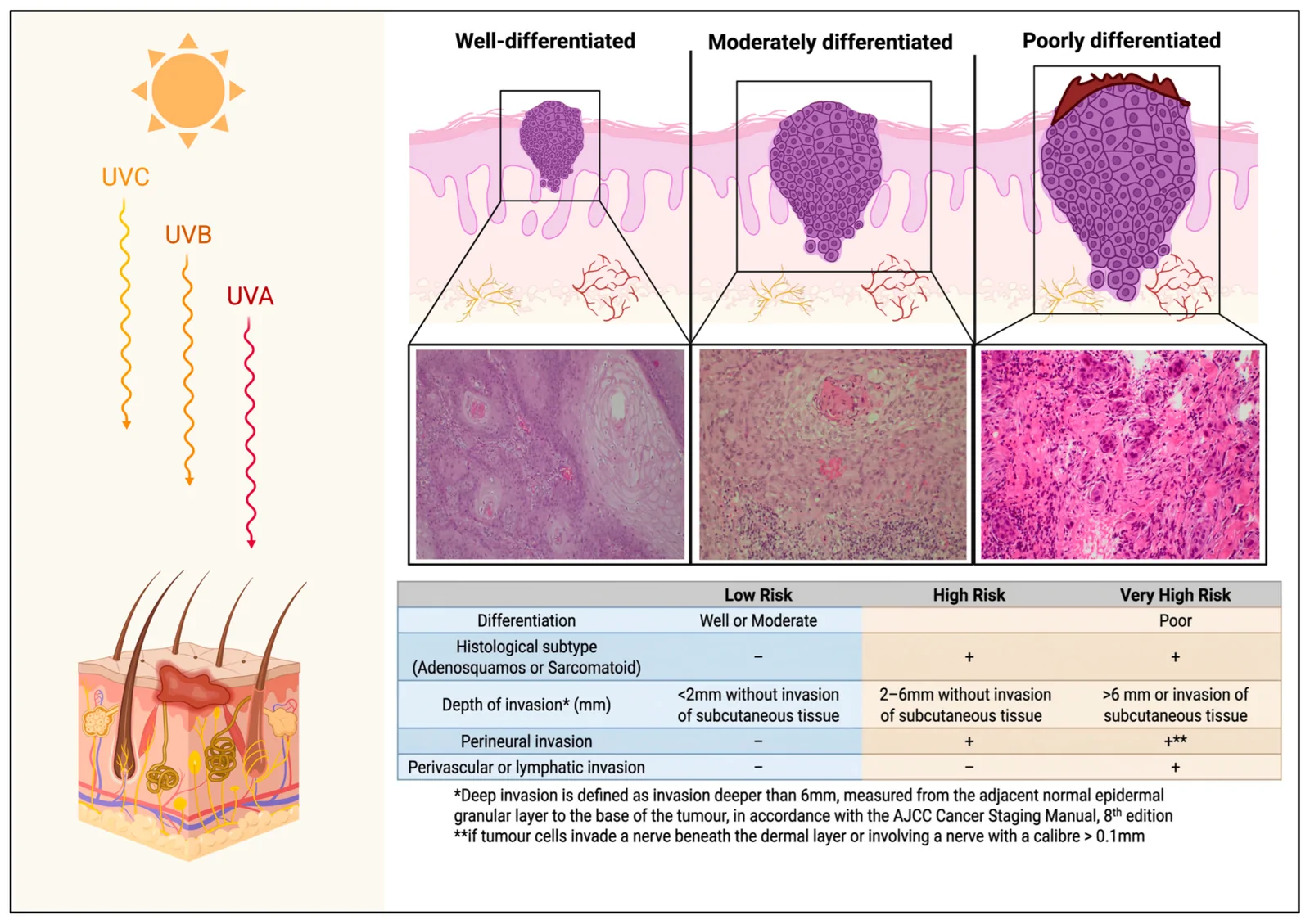
Adapted form of risk classification of cSCC, illustration by NCCN (created in BioRender; Manole, M. (2026) https://BioRender.com/nw7kben) (URL accessed on 26 June 2026) [34].

The treatment of cSCC is primarily surgical, with electrodesiccation and curettage used for in situ lesions and Mohs surgery or wide local excision for invasive tumours, particularly those with high-risk features [24,31]. For advanced or inoperable cases, systemic therapies have been developed [24,31]. Traditional chemotherapeutic agents such as doxorubicin, cisplatin, and paclitaxel have demonstrated efficacy but are limited by their toxicity [24]. More recently, immunotherapy with programmed cell death protein 1 (*PD-1*) inhibitors, including cemiplimab and pembrolizumab, has become the preferred systemic treatment for advanced disease due to durable responses and improved tolerability [24,31]. Targeted therapy with epidermal growth factor receptor (*EGFR*) inhibitors, such as cetuximab, represents an alternative option for patients who are not candidates for immunotherapy [32]. Future therapeutic approaches may include histone deacetylase inhibitors such as vorinostat, remetinostat, and abexinostat, which are currently under investigation [24,31]. Overall, optimal management of complex cSCC cases relies on a multidisciplinary approach and continued therapeutic advances [24,31]. Figure 1 provides a summary of the major risk factors and the key genetic alterations involved in the pathogenesis of cSCC [33] whereas Figure 2 presents an adapted illustration of the NCCN risk classification system for cSCC [34].

The novelty of this study lies in its epidemiological relevance, as it represents one of the largest retrospective Romanian cohorts of cSCC analyzed to date. The study integrates epidemiological data with histopathological, topographic, and volumetric assessments, providing a better understanding of tumour aggressiveness, risk stratification, and surgical outcomes.

In addition, the inclusion of cases from the pre-COVID-19, COVID-19, and post-COVID-19 periods allows for the assessment of temporal variations in diagnostic patterns, healthcare accessibility, and disease presentation. Although a direct causal relationship between COVID-19 and cSCC could not be established, the observed epidemiological shifts could highlight the potential role of infectious events and diseases in modulating cancer presentation and progression. These findings emphasize the importance of incorporating emerging and non-traditional risk factors, such as pandemics, viral infections, and other systemic pathogenic influences, into future epidemiological research to improve our understanding of skin cancer development and risk stratification. Although previous studies have investigated the epidemiology of cSCC, few have comprehensively evaluated the impact of the COVID-19 pandemic on the clinical and histopathological characteristics of tumours, risk stratification, surgical management, and tumour volumes, particularly in Eastern European populations.

The aim of the study was to investigate the epidemiological, clinical, histopathological, and surgical characteristics of cutaneous squamous cell carcinomas diagnosed at the Mureș County Clinical Hospital, and to identify tumour-related pathological factors associated with biological aggressiveness and risk stratification within one of the largest Romanian cSCC cohorts. 

## 2. Materials and Methods

This was a retrospective, descriptive, and observational study conducted by analyzing data obtained from histopathological reports, as well as by examining histological slides and immunohistochemistry (IHC) reactions of patients diagnosed with cSCC within the Clinical Pathology Department of the Mureș Clinical County Hospital, between January 2017 and December 2025. A total of 332 lesions were included, originating from both surgical and non-surgical departments.

### 2.1. Inclusion and Exclusion Criteria

The primary inclusion criterion was the histopathological diagnosis of cSCC, established by microscopic examination of the lesions, for tissue samples received between January 2017 and December 2025. Other histopathological diagnoses were excluded, as were all lesions outside the predefined study period. Additionally, cases of cSCC with a non-cutaneous origin were excluded, together with those that represented metastases from a primary tumour located in another organ.

### 2.2. Data Collection and Database Organization

Data collection was performed using Microsoft Excel for iOS v.16.103.3 (Microsoft Corporation, Redmond, WA, USA).The collected epidemiological data included: the year of diagnosis, demographic data (age, gender, and environment of origin), anatomical site of excision, shape of the excised specimen, and dimensions of both the excised specimens and the tumours. Information regarding the patients’ environment of residence was available in 272 of the 332 included cases.

Anatomical sites were classified into regions as follows:

- Head and neck: Scalp, facial, frontal, auricular, ocular, nasal, and cervical.
- Thorax: Trunk, pectoral, mammary, sub-, and supraclavicular.
- Abdomen: All cases with abdominal localization.
- Anogenital: Labia majora, vulva, genital, penile, perianal, perineal, and sacral.
- Unspecified: Cases in which the excision site is not documented.

The grouping of excision sites into regions allowed for a reduction in categories without loss of clinically relevant information and facilitated the application of statistical tests. Tumoral and specimen excision volumes were calculated in mm^3^ according to the following formula: length (mm) × width (mm) × height (mm). When height measurements were unavailable, an arbitrary value of 1 mm was assigned, and all lesions that presented only one dimension were excluded from the volumetric analysis. From the total of 332 lesions, the volume was successfully calculated for 299 tumours and 331 excised specimens. The term *tumour* refers to the neoplastic lesion itself, whereas *excised specimen* refers to the entire surgically removed tissue sent for histopathological examination, including the tumour and the surrounding non-neoplastic tissue.

The data regarding cSCC characteristics were as follows: keratinization, degree of differentiation, histological subtypes, presence or absence of ulceration, infiltration, and depth of invasion. These parameters were required to assign each lesion to a risk category according to the U.S. National Comprehensive Cancer Network (NCCN) guidelines for risk stratification, which are used to guide treatment based on the risk of recurrence, metastasis, or disease-related mortality. Pathological criteria used for risk stratification in this study were derived from the “Pathology” section of the NCCN guidelines, and lesions were classified according to the scheme presented below, adapted from NCCN version 1.2026.

For the temporal analysis, the cohort was divided into three main study periods according to the date of diagnosis as follows:

- Pre-COVID-19: All cases diagnosed before the COVID-19 national emergency decree came into effect, including cases diagnosed in 2017, 2018, and 2019.
- COVID-19: All cases diagnosed during the COVID-19 emergency decree, including all cases diagnosed from 2020 until the 7th of March 2022.
- Post-COVID-19: All cases diagnosed after the lifting of restrictions under the COVID-19 emergency decree, including all cases from the 8th of March 2022 until December 2025.

### 2.3. Statistical Analysis

Statistical analysis was performed using Microsoft Excel for iOS v.16.103.3 (Microsoft Corporation, Redmond, WA, USA). and GraphPad Prism version 11 (GraphPad Software LCC, Boston, MA, USA). Data distribution was assessed by evaluating kurtosis and skewness parameters, inspection of histograms and Q-Q plots, and by applying the Shapiro–Wilk test, all of which confirmed a non-parametric distribution.

Quantitative variables were expressed as the median (interquartile range, [IQR]), while qualitative variables were presented as absolute and relative frequencies. For comparing the qualitative data, the Mann–Whitney test and Kruskal–Wallis test were used as appropriate, and the Spearman correlation test was used for correlation analyses.

**Figure 3:**
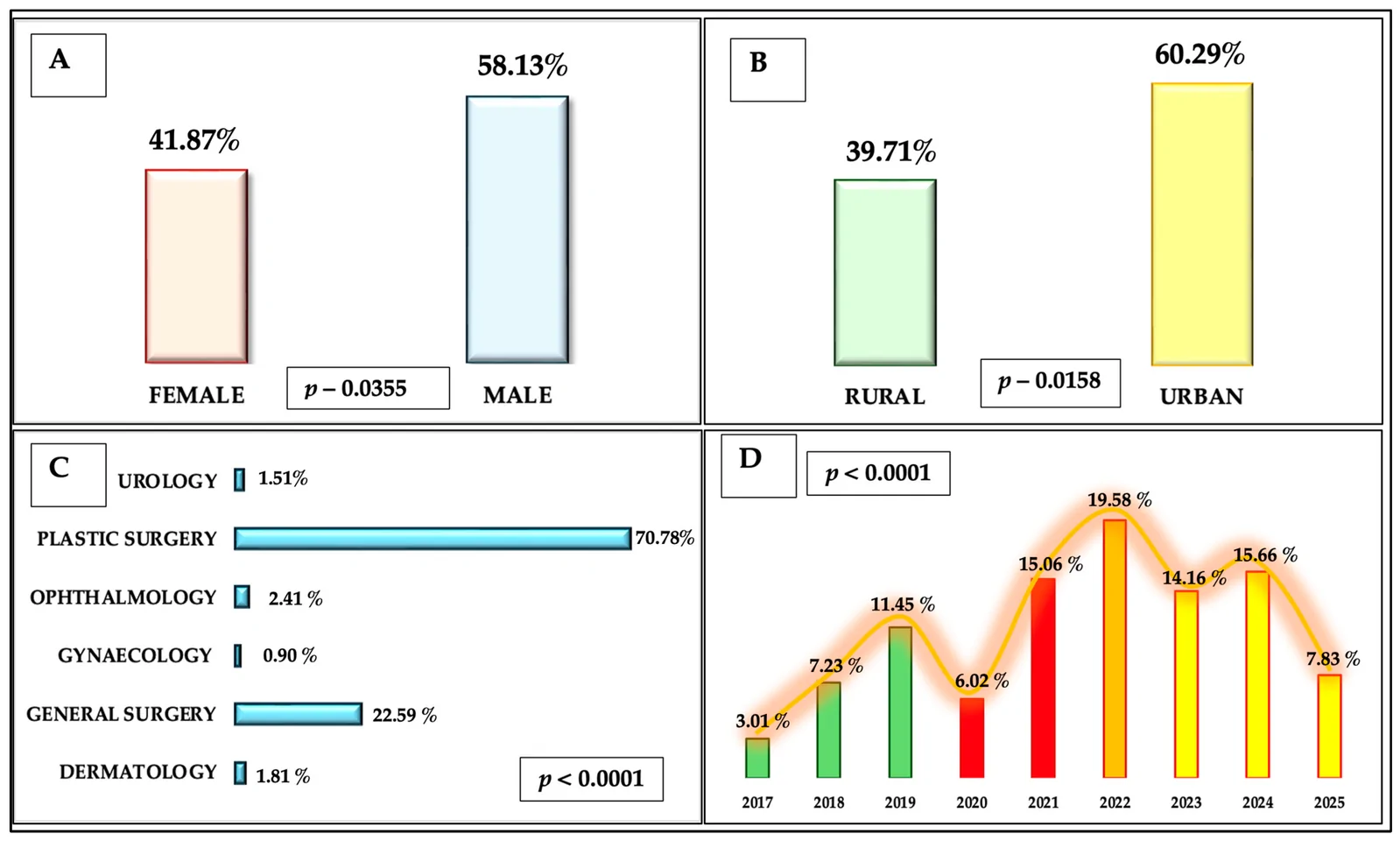
Data distribution across the cohort and study years: (**A**) presents gender distribution; (**B**) presents the environment of origin; (**C**) is the department where patients underwent excision; (**D**) is the study period divided into pre-COVID-19 in green and COVID-19 in red. In accordance with the study design, 2022 was divided according to the date on which national authorities declared the end of the COVID-19 state of alert; therefore, year 2022 is depicted in a different orange. The post-COVID-19 period is in yellow.

For qualitative variables, Fisher’s exact and Chi-square tests (with Yates correction or standard) were applied according to the situation. In specific situations, theoretical *p*-values were calculated under the assumption of equal distribution of data between groups (as seen in Figure 3).

The threshold for statistical significance was set at *α* ≤ 0.05, and both statistically significant results and values exceeding this threshold were reported.

### 2.4. Ethical Considerations

This study was conducted in accordance with the principles of the Declaration of Helsinki, and was approved by the Institutional Ethics Committee of the Mureș County Clinical Hospital in Târgu Mureș under approval number 18922 from 26 January 2026.

## 3. Results

### 3.1. General Characteristics and Demographics of the Cohort

The study included a total of 332 lesions diagnosed in patients aged between 36 and 98 years. Figure 3 summarizes the main demographic characteristics of the cohort, including gender distribution, area of residence (environment of origin), the department where the specimens originated and the year of diagnosis. Table 1 aims to analyze the distribution of cases according to the age of the cohort.

**Table 1:** Distribution of cases and median age analysis of the cohort according to epidemiological factors.

Variable	n	Q1	Median (Age)	Q3	*p*
Total	332	69	76	83	N/A *
Female	139	71	77	83	0.0489
Male	193	67	75	83	
Rural	108	68	77	83	0.7418
Urban	164	69	76	83	

* N/A—not assessed.

### 3.2. Anatomical Distribution

Table 2, Table 3 and Table 4 present the distribution of lesions according to the anatomical site of excision. The analysis included both the overall distribution of cases based on excised specimens and a more detailed evaluation of lesions located in the head and neck region.

### 3.3. Classification of Tumours, Aggressiveness, and Histopathological Characteristics

Table 5 illustrates the distribution of cases according to tumour risk subclasses. The anatomical distribution of lesions, including the head and neck subregions, according to the excision site, is detailed in Table 6 and Table 7, while Table 8 presents the age analysis in relation to risk class. Table 9 examines tumour risk class distribution according to gender and environment of origin. The histopathological characteristics presented in Table 10 show cSCC differentiation in relation to demographic, anatomical, and temporal factors. Table 11 shows the distribution of tumour differentiation degrees at different excision sites outside the head and neck region. Additionally, the association between tumour risk and the presence of ulceration is shown in Table 12, while Table 13 presents the relationship between ulceration and different study periods.

### 3.4. Temporal Analysis in Relation to the Pre-COVID-19, During COVID-19 and Post-COVID-19 Periods

In this section, the epidemiological and histopathological characteristics of cSCC were analyzed over time in relation to the pre-COVID-19, COVID-19, and post-COVID-19 periods. Table 14 presents the temporal analysis of demographic characteristics, risk group variations, tumour behaviour and recurrence rates across these periods. Table 15 focuses on the third COVID-19 wave, associated with the delta variant, known for its high transmissibility, with an analysis of tumour aggressiveness during the wave, and at 6 months and 1 year.

### 3.5. Surgical Outcomes and Macroscopic Characteristics

This subsection summarizes the surgical outcomes and macroscopic characteristics of excised specimens. Resection margin status was evaluated according to the study period and tumour risk grades, as shown in Table 16 and Table 17. The macroscopic characteristics of both tumours and specimens, including the shape and association with inflammatory infiltrate, were analyzed in relation to tumour risk. The relationship between macroscopic appearance and margin infiltration is presented in Table 18, while Table 19 examines macroscopic tumour appearance in relation to inflammatory infiltrate and the different risk categories.

### 3.6. Volumetric Analysis

In this subsection, Table 20 and Table 21 present an analysis the of volumes of both excised specimens and tumours in order to quantify tumour extent and to evaluate their relationship with demographic, histopathological, and temporal parameters. Tumour volumes were also assessed in relation to risk, the presence of ulceration, resection margin status, and the occurrence of recurrences. In addition, variations in volume according to the diagnostic period were examined, along with correlations between volume, patient age, and year of diagnosis.

## 4. Discussion

The demographic analysis within Figure 3 revealed a significant male predominance within the cohort, with men accounting for 58.1% of cases compared to 41.9% among women (*p* = 0.0335) [35,36,37,38]. Regarding age distribution within Table 1, a significant gender-related difference was observed, with women presenting a higher median age (77; IQR [71–83]) at diagnosis compared to men (75; IQR [67–83]) [35]. These findings are consistent with previously published epidemiological studies on cSCC and other non-melanoma skin cancers, which report a higher incidence among men and disease onset at older ages, largely attributed to the cumulative effects of chronic exposure to UV radiation [37,38]. The observed difference in median age may reflect variations in occupational sun exposure, as well as individual photoprotective behaviours [35,36,37].

Analysis based on the environment of origin (Figure 3) demonstrated a significantly higher number of cases (*p* = 0.0158) from urban areas (60.30%) compared to rural areas (39.70%). This difference is more likely related to limited access to medical services among individuals living in rural areas, in contrast to the relatively easier access available in urban settings [39,40]. These findings also align with national studies reporting increasing disparities in healthcare accessibility for people living in rural areas [2,3].

The temporal analysis within Figure 3 showed a significant variation in case distribution across the pre-COVID-19, COVID-19, and post-COVID-19 periods (*p* < 0.0001), with a relative reduction in diagnosed cases during the pandemic and a subsequent increase in the post-pandemic interval. These changes appear to reflect altered healthcare access and patient presentation behaviour during the pandemic, a phenomenon also reported in studies evaluating the impact of COVID-19 on oncological and dermatological disease diagnosis and management [38,39].

From a topographic perspective, the anatomical distribution presented in Table 2 demonstrated a predominant localization in the head and neck region, accounting for 65.06% of all cases (*p* < 0.0001). This distribution reconfirms the major role of chronic UV exposure in cSCC pathogenesis, as effective photoprotective measures are harder to implement in this area [40,41,42,43,44,45], even though no significant gender-related differences (Table 2) were observed with regard to the overall anatomical localization (*p* = 0.2954), suggesting a similar topographic pattern of occurrence between men and women. However, a significant difference according to the environment of origin (Table 2) was identified (*p* = 0.0480), with head and neck localization being more frequent among patients from rural compared to urban areas. This may reflect prolonged occupational sun exposure and reduced use or accessibility of photoprotective measures in rural populations [2,40,46].

A detailed analysis of the head and neck anatomical subsites presented within Table 3 demonstrated a non-uniform distribution, with a predominance of facial (36.1%) and auricular (24.5%) lesions (*p* < 0.0001). For auricular lesions, it was first reported in 1991 by the Social and Preventive Medicine Department at the University of Queensland Medical School that more than half of the participants neglected the auricular area while applying sunscreen products [47]. Significant gender-related differences were also observed (*p* < 0.0001), with men more frequently presenting auricular and scalp lesions, while women predominantly exhibited facial involvement [48,49,50]. These differences may be explained by facial hair patterns in men, traditional headscarves, and ear coverage among women in Balkan populations, or the protective effect of long hair against UV exposure in the auricular region [49,50]. No significant differences were identified in median age according to excision site (*p* = 0.1391), despite a lower median age observed for anogenital excision (73 years; IQR [65.50–76.25]) and an older median age in upper limb excisions (82 years; IQR [71–84]) (Table 4).

Regarding tumour risk within Table 5, most lesions were classified as low risk (LR) (62.35%; *p* < 0.0001), followed by high risk (HR) (19.88%), and very high risk (VHR) (17.77%). Head and neck lesions represented the predominant anatomical site in all three risk groups, whereas the relative distribution of tumours at other locations varied according to risk class. Specifically, we found that high-risk tumours were proportionally more frequent on the upper limbs, thorax, and anogenital region, while very-high-risk tumours showed a relatively greater representation on the lower limbs. Although this association is expected because anatomical location constitutes one of the parameters included in the NCCN risk stratification system, our findings confirm that the distribution of risk categories within this cohort is consistent with current guideline-based classification [51,52]. These observations further support the relevance of tumour topography as an important component of risk assessment in cSCC, particularly for anatomical sites recognized as carrying an inherently higher risk of adverse outcomes, such as the face, ears, genitalia, hands, feet, ankles, and nipples/areolae [39,51,53]. A detailed sublocalization analysis within Table 7 revealed that lesions occurring predominantly within the facial region of the head and neck were significantly associated (*p* = 0.0314) with HR (42.42%) and VHR (39.16%), findings that are consistent with other studies [40,54], even though most cSCCs were classified as LR. No significant association was identified between tumour risk class and age (*p* = 0.7853), gender (*p* = 0.2948), or environment of origin (*p* = 0.9186) (Table 8 and Table 9), supporting the idea that cSCC aggressiveness is determined predominantly by histopathological and topographic characteristics rather than isolated demographic factors [24,40,48,49].

From a histopathological perspective, the tumour differentiation presented within Table 10 was significantly influenced by the environment of origin (*p* = 0.0083) and anatomical site (*p* < 0.0001), with poorly differentiated lesions occurring more frequently outside the head and neck area, particularly in the lower limb (41.18%), and equally in the thorax (25.53%) and upper limb (25.53%) regions. Recent research has shown that women are more prone to develop cSCC of the lower limb, and that localisation other than head and neck suggests a different aetiology, which could result in different tumour behaviour [18,24]. More precisely, extracranial regions have a higher probability of inflammation-related lesions, such as ulcers, trauma, and prior irradiation, all resulting in higher proportions of highly aggressive tumours [18,24].

Temporal analysis (Table 10) of the differentiation grade revealed significant differences across the analyzed periods (*p* = 0.0349), with well-differentiated lesions predominating before and after the pandemic, and an increased number of poorly differentiated cSCCs observed during the COVID-19 period [55,56,57,58,59,60]. These findings suggest temporary variations in the histopathological profile, potentially related to reduced access to medical services during the pandemic or to a possible influence of SARS-CoV-2 infection on immune function through induced immunosuppression or treatment effects. However, no direct causal relationship can be established, and the observed association appears to be temporal rather than causal [55,56,57,58].

Furthermore, the analysis of tumour differentiation in anatomical regions outside the head and neck area (Table 11) revealed a statistically significant variation in differentiation patterns according to excision site (*p* = 0.0037). Poorly differentiated tumours were predominantly observed in the lower limbs, while upper limb lesions were more frequently classified as moderately or well-differentiated cSCCs. Even though there is no evidence to support limb distribution differentiation, many articles still provide evidence supporting the association between specific anatomical distribution and aggressiveness [24,31,54,61]. As shown in Table 12, ulceration was significantly associated with tumour risk class (*p* < 0.0001), with ulcerated lesions being more frequently classified as VHR (66.10%) [24,56,57]. In contrast, the ulceration distribution shown in Table 13 did not differ significantly between the pre-COVID-19, COVID-19, and post-COVID-19 periods (*p* = 0.5574), suggesting that ulceration reflects intrinsic tumour characteristics rather than temporal contextual influences [24,56,57].

The temporal cohort analysis within Table 14 demonstrated that the distribution of major demographic variables remained relatively stable across the analyzed periods, with no significant differences in gender (*p* = 0.7279) or environment of origin (*p* = 0.3205). Similarly, tumour risk class distribution (Table 14) did not differ significantly between the analyzed periods (*p* = 0.7992), indicating that the pandemic did not influence major shifts in the overall tumour risk profile. However, HR and VHR lesions showed a post-COVID-19 increase compared to the COVID-19 period [57,58,59,60], with a non-significant trend towards increased infiltrative behaviour, as presented in Table 14, during the post-COVID-19 period (*p* = 0.0775). Recurrence rates remained relatively stable across periods (*p* = 0.8014), suggesting that the pandemic exerted only a short-term impact on oncological outcomes within the cohort.

Table 15 presents a detailed analysis of the Delta variant of SARS-CoV-2 virus during July to October 2021 compared with 6-month and 12-month follow-up intervals. The analysis revealed no significant difference (*p* = 0.4025) regarding tumour risk class distribution, indicating no persistent increase in tumour aggressiveness following this severe pandemic wave. Therefore, a temporal association cannot be proven at this point, even though it is well established in the literature that viruses can be oncogenic, induce immunosuppression and constitute a risk factor in the pathogenesis of cSCC [52,58,59].

Surgical outcome analysis (Table 16) demonstrated that resection margin status did not vary significantly according to the diagnostic period (*p* = 0.9596), with tumour-free margins remaining stable before, during, and after the pandemic. These findings suggest that, despite pandemic-related constraints, oncological surgical standards were maintained [52,58,59]. In contrast, margin status was significantly associated with tumour risk class (*p* = 0.0014), with VHR lesions showing a substantially higher rate of positive margins (40.68%) compared to HR (21.21%) and LR (18.36%) lesions (Table 17), highlighting the difficulty of achieving complete excision in biologically aggressive tumours with subclinical extension [60,61]. Macroscopically, excision specimens (Table 18) were predominantly elliptical (59.64%), followed by irregular shapes (32.23%) (*p* < 0.0001). Elliptical excisions (Mohs excision) were significantly more likely to achieve tumour-free margins compared to irregular excisions (*p* = 0.0085), supporting their technical advantage and reinforcing their role as the standard surgical approach in oncologic and reconstructive surgery [62,63]. Regarding the tumour macroscopic characteristics from Table 19, lesions were significantly more frequently irregular (64.71%), followed by nodular (28.43%) and round-oval (8.86%) forms (*p* < 0.0001). However, the presence of inflammatory infiltrate was not significantly associated with macroscopic shape (*p* = 0.9910) or tumour risk class (*p* = 0.4407), suggesting that this parameter reflects a local host response rather than a direct indicator of tumour aggressiveness [8,31,64,65].

Volumetric analysis within Table 20 revealed a wide variability in both excision specimen and tumour volume, reflecting the clinical and biological heterogeneity of cSCC. Excision specimen volumes were significantly larger in VHR lesions (median: 2880 mm^3^; IQR [720–11,200 mm^3^]) compared with HR (median: 717.5 mm^3^; IQR [260.5–3411 mm^3^]) and LR lesions (median: 1766 mm^3^; IQR [630–4530 mm^3^]; *p* = 0.0004). A similar association was observed with ulceration, with excision specimens from ulcerated lesions showing significantly larger volumes (median: 2520 mm^3^; IQR [840–7020 mm^3^]) compared to non-ulcerated lesions (median: 1075 mm^3^; IQR [394.8–2690 mm^3^]; *p* < 0.0001).

Specimen volumes were not influenced by gender (*p* = 0.5221), environment of origin (*p* = 0.7665), margin status (*p* = 0.9275), or recurrence (*p* = 0.1371), indicating that excision size is primarily determined by intrinsic tumour characteristics and clinical and histopathological features rather than demographics [45]. Additionally, no significant differences were observed in specimen volumes across the study period, suggesting a consistent surgical approach over time [52,58,59].

Tumour volumes showed similar findings, with VHR lesions exhibiting significantly larger volumes (median: 255 mm^3^; IQR [70–1020 mm^3^]; *p* = 0.0070). Ulcerated tumours also showed significantly increased volumes (median: 268 mm^3^; IQR [75–1103 mm^3^]; *p* = 0.0003), reinforcing ulceration as a marker of biological aggressiveness. Interestingly, reoccurrences were associated with significantly smaller tumour volumes at initial excision (median, 60 mm^3^; IQR [18–120 mm^3^] vs. 200 mm^3^; IQR [54–888.8 mm^3^]; *p* = 0.0360), potentially reflecting distinct biological behaviour or the limitations of initial clinical volumetric assessment in predicting subsequent tumour evolution or even accurate assessment of tumour depth [24,49,66,67,68,69,70].

Temporally, tumour volume varied significantly across periods (*p* < 0.0001), with larger median volumes observed during the COVID-19 period (Table 20) compared with the pre- and post-pandemic intervals, suggesting delayed presentation or diagnosis under pandemic restrictions [53,54,68]. Correlation analysis showed a significant positive correlation between excision specimen volume and patient age (*r* = (+)0.21; *p* = 0.0001; Table 21), indicating that larger specimens were frequently obtained from older patients. Tumour volume showed similar results, suggesting that older patients tended to present with larger tumours as well (*r* = (+)0.22; *p* = 0.0001). In contrast, excision specimen volume showed no significant correlation with the year of diagnosis (*r* = (−)0.058; *p* = 0.2898). However, tumour volume showed a significant negative correlation with the year of diagnosis (*r* = (−)0.23; *p* < 0.0001), indicating a temporal trend towards the diagnosis of smaller tumours in more recent years, possibly reflecting improved healthcare access and earlier detection following the pandemic period, with similar reports described in other NMSC cohorts [2,71,72,73,74,75,76]. Patient age, although showing a weak positive correlation with the year of diagnosis (*r* = (+)0.13; *p* = 0.0190), suggested a modest increase in the age of patients diagnosed during the study period. This could probably be explained by the serious difficulties experienced by the Romanian medical system during the COVID-19 pandemic but also in the years right after the end of the pandemic, resulting in delayed presentations [2,71,72,73,74,75,76].

### Study Limitations

This study has several limitations. As a retrospective study, variables of interest and confusion cannot be controlled. Although it represents one of the largest Romanian cSCC cohorts to date, it may still be subject to geographical bias and some demographic, histopathologic and anatomical variants may be underrepresented. Volumetric analysis was estimated from the available macroscopic dimensions and should therefore be considered approximate, as the calculations were based on arbitrary values in cases where only two dimensions were available. Furthermore, we have no data regarding SARS-CoV-2 infection status nor exposure to other occupational risk factors.

## 5. Conclusions

This retrospective study provides a comprehensive analysis of the demographic, clinical, histopathological, and temporal characteristics of cutaneous squamous cell carcinoma (cSCC), highlighting tumour intrinsic features as the primary determinants of biological aggressiveness. The study population was predominantly elderly, with a clear male predominance and a lower median age compared with women, consistent with known epidemiological patterns for cSCC and other non-melanoma skin cancers. Tumoral location emerged as an important determinant of biological aggressiveness, with most lesions arising within the head and neck area and showing a strong association with high- and very-high-risk categories. The heterogeneous distribution of sublocalizations and observed gender-related differences likely reflect cumulative ultraviolet exposure and behavioural factors. Tumour differentiation demonstrated a distinct anatomical pattern, as poorly differentiated lesions occurred more frequently outside the head and neck region, suggesting potential differences in tumour biology according to anatomical sites.

In contrast, demographic characteristics such as age and environment of origin did not significantly influence risk class distribution, supporting the idea that cSCC aggressiveness is driven primarily by histopathological and anatomical characteristics rather than isolated demographic factors.

The COVID-19 pandemic mainly affected diagnostic timing and healthcare access, resulting in a temporary reduction in cases and variations in differentiation grade, without sustained effects on risk profiles, infiltrative behaviour, or recurrence rates.

Surgical outcomes remained stable across the periods, indicating preserved oncological standards, although very-high-risk tumours were more frequently associated with positive resection margins. Ulceration was consistently associated with very-high-risk lesions and increased tumour volume, reinforcing its importance as an indicator of tumour aggressiveness. Volumetric analysis further supported tumour and excision specimen volumes as indirect markers of aggressiveness, given their significant association with risk class and ulceration status. Although larger tumours were observed in elderly patients during the pandemic period, the overall trend toward smaller tumour sizes in recent years suggests improved early detection following the resolution of pandemic-related healthcare constraints, even though patients were referred at increasingly older ages.

Overall, these findings underscore that cSCC biological behaviour is predominantly determined by tumour-specific, histopathological, and topographical features, while demographic and contextual factors mainly influence diagnostic timing and access to care, emphasizing the need for refined risk stratification and continued efforts toward earlier detection and optimized surgical management.

## Figures and Tables

**Table 2:** Anatomical distribution of excision sites by gender and environment of origin.

	Head and Neck	Upper Limb	Lower Limb	Thorax	Anogenital	Other	Abdomen	*p*
Total	216 (65.06%)	51 (15.36%)	17 (5.12%)	22 (6.63%)	14 (4.22%)	8 (2.41%)	4 (1.20%)	N/A *
Female	82 (58.99%)	22 (15.83%)	10 (7.19%)	11 (7.91%)	8 (5.76%)	5 (3.60%)	1 (0.72%)	0.2954
Male	134 (69.43%)	29 (15.03%)	7 (3.63%)	11 (5.70%)	6 (3.11%)	3 (1.55%)	3 (1.55%)	
Rural	84 (77.78%)	13 (12.04%)	3 (2.78%)	2 (1.85%)	3 (2.78%)	2 (1.85%)	1 (0.93%)	0.0480
Urban	100 (60.98%)	26 (15.85%)	12 (7.32%)	14 (8.54%)	8 (4.88%)	2 (1.22%)	2 (1.22%)	

* N/A—not assessed.

**Table 3:** Distribution of head and neck cases by excision site.

	Facial	Labial	Ocular	Nasal	Scalp	Cervical	Auricular	*p*
Total	78 (36.11%)	10 (4.63%)	19 (8.79%)	20 (9.25%)	25 (11.57%)	11 (5.09%)	53 (24.53%)	N/A *
Female	45 (54.88%)	2 (2.44%)	11 (13.41%)	11 (13.41%)	5 (6.10%)	4 (4.87%)	4 (4.87%)	<0.0001
Male	33 (24.62%)	8 (5.97%)	8 (5.97%)	9 (6.72%)	20 (14.93%)	7 (5.22%)	49 (36.57%)	
Urban	40 (40.00%)	1 (1.00%)	11 (11.00%)	9 (9.00%)	17 (17.00%)	2 (2.00%)	20 (20.00%)	0.0134
Rural	31 (36.90%)	7 (8.33%)	4 (4.76%)	7 (8.33%)	6 (7.14%)	7 (8.33%)	22 (26.19%)	

* N/A—not assessed.

**Table 4:** Median age distribution according to excision sites.

Excision Area	n (%)	Q1 (Years)	Median (Years)	Q3 (Years)	*p*
Head and neck	216 (65.06%)	69	76	82	0.1391
Upper Limb	51 (15.36%)	71	82	84	
Lower Limb	17 (5.12%)	64	76	81	
Thorax	22 (6.63%)	67	78	86	
Anogenital	14 (4.22%)	66	73	76	
Other	8 (2.41%)	68	72	81	
Abdomen	4 (1.20%)	55	77	79	

**Table 5:** Distribution of cases according to the subclass.

Risk Class	n (%)	*p*
LR	207 (62.35%)	<0.0001
HR	66 (19.88%)	
VHR	59 (17.77%)	

LR—low risk, HR—high risk, VHR—very high risk.

**Table 6:** Distribution of lesions according to their anatomical excision sites.

Risk	Head and Neck	Upper Limb	Lower Limb	Thorax	Anogenital	Other	Abdomen	*p*
VHR	40 (67.80%)	4 (6.78%)	7 (11.86%)	4 (6.78%)	2 (3.39%)	1 (1.69%)	1 (1.69%)	0.0051
HR	33 (50.00%)	10 (15.15%)	3 (4.55%)	8 (12.12%)	6 (9.09%)	4 (6.06%)	2 (3.03%)	
LR	143 (69.08%)	37 (17.87%)	7 (3.38%)	10 (4.83%)	6 (2.90%)	3 (1.45%)	1 (0.48%)	

LR—low risk, HR—high risk, VHR—very high risk.

**Table 7:** Distribution of lesions according to their excision site in the head and neck region.

Risk	Facial	Labial	Ocular	Nasal	Scalp	Cervical	Auricular	*p*
VHR	6 (15.00%)	3 (7.50%)	4 (10.00%)	5 (12.50%)	3 (7.50%)	2 (5.00%)	17 (42.50%)	0.0314
HR	12 (36.36%)	2 (6.06%)	4 (15.15%)	0 (0.00%)	3 (18.18%)	3 (9.09%)	5 (15.15%)	
LR	48 (33.57%)	5 (3.50%)	10 (6.99%)	15 (10.49%)	28 (19.58%)	6 (4.20%)	31 (21.68%)	

LR—low risk, HR—high risk, VHR—very high risk.

**Table 8:** Risk class distribution according to age median.

Risk Class	n	Q1 (Years)	Median (Years)	Q3 (Years)	*p*
VHR	59	70	76	81	0.7853
HR	66	68	76	82	
LR	207	69	76	83	

LR—low risk, HR—high risk, VHR—very high risk.

**Table 9:** Risk class distribution according to gender and environment of origin.

Risk	VHR	HR	LR	*p*
Female	22 (15.83%)	33 (23.74%)	84 (60.43%)	0.2948
Male	37 (19.17%)	33 (17.10%)	123 (63.73%)	
Rural	21 (19.44%)	19 (17.59%)	68 (62.96%)	0.9186
Urban	32 (19.51%)	32 (19.51%)	100 (60.98%)	

LR—low risk, HR—high risk, VHR—very high risk.

**Table 10:** Distribution of differentiation in relation to demographic, anatomical, and temporal factors.

cSCC Differentiation		Poor	Medium	Well	*p*
Gender	Male	22 (50.00%)	61 (67.03%)	76 (57.58%)	0.1357
	Female	22 (50.00%)	30 (32.97%)	56 (42.42%)	
Origin	Rural	11 (28.95%)	35 (48.61%)	41 (60.29%)	0.0083
	Urban	27 (71.05%)	37 (51.39%)	27 (39.71%)	
Area	Head and neck	25 (22.73%)	69 (75.82%)	88 (68.22%)	<0.0001
	Other areas	17 (77.27%)	22 (24.18%)	41 (31.78%)	
Temporal	pre-COVID-19	9 (16.98%)	22 (41.51%)	22 (41.51%)	0.0349
	COVID-19	16 (21.33%)	15 (20.00%)	44 (58.67%)	
	post-COVID-19	18 (13.04%)	54 (39.13%)	66 (47.83%)	

**Table 11:** Tumour differentiation degrees in excision sites other than the head and neck regions.

cSCC Differentiation	Thorax	Abdomen	Upper Limb	Lower Limb	Anogenital	*p*
Poor	4 (23.53%)	1 (5.88%)	4 (23.53%)	7 (41.18%)	1 (5.88%)	0.0037
Moderate	3 (13.64%)	1 (4.55%)	11 (50.00%)	1 (4.55%)	6 (27.27%)	
Well	7 (17.07%)	1 (2.44%)	26 (63.41%)	6 (14.63%)	1 (2.44%)	

**Table 12:** Association between tumour risk and ulcerations.

	VHR	HR	LR	*p*
Ulcerated	39 (66.10%)	19 (28.79%)	110 (53.14%)	<0.0001
Non-Ulcerated	20 (33.90%)	47 (71.21%)	97 (46.86%)	

LR—low risk, HR—high risk, VHR—very high risk.

**Table 13:** Association between ulceration and different periods.

	Pre-COVID-19	COVID-19	Post-COVID-19	*p*
Ulcerated	37 (51.39%)	46 (54.76%)	84 (47.73%)	0.5574
Non-Ulcerated	35 (48.61%)	38 (45.24%)	92 (52.27%)	

**Table 14:** Distribution of demographic, clinical and histopathological characteristics according to the COVID-19 study period.

		Pre-COVID-19	COVID-19	Post-COVID-19	*p*
Gender	Female	33 (45.83%)	35 (41.67%)	71 (40.34%)	0.7279
	Male	39 (54.17%)	49 (58.33%)	105 (59.66%)	
Environment	Rural	12 (46.15%)	22 (32.84%)	74 (42.53%)	0.3205
	Urban	14 (53.85%)	45 (67.16%)	100 (57.47%)	
Risk Group	VHR	12 (16.67%)	8 (11.11%)	26 (14.77%)	0.7992
	HR	18 (25.00%)	15 (20.83%)	39 (22.16%)	
	LR	42 (58.33%)	49 (68.06%)	111 (63.07%)	
Tumoral Behaviour	Infiltrative	49 (68.06%)	53 (63.10%)	134 (76.14%)	0.0775
	Non-infiltrative	23 (31.94%)	31 (36.90%)	42 (23.86%)	
Recurrence	Recurrence	3 (4.69%)	6 (7.14%)	9 (5.11%)	0.8014
	Non-recurrence	61 (95.31%)	78 (92.86%)	167 (94.89%)	

LR—low risk, HR—high risk, VHR—very high risk.

**Table 15:** Third wave (delta Δ): analysis of aggressiveness during the wave and at 6-month and 1-year follow-up.

Risk Class	July–October 2021	January–April 2022	July–October 2022	*p*
LR	13 (61.90%)	20 (80.00%)	13 (65.00%)	0.4025
HR	3 (14.29%)	2 (8.00%)	5 (25.00%)	
VHR	5 (23.81%)	3 (12.00%)	2 (10.00%)	

LR—low risk, HR—high risk, VHR—very high risk.

**Table 16:** Temporal analysis of resection margins.

Resection Margins	Pre-COVID-19	COVID-19	Post-COVID-19	*p*
Free	55 (76.39%)	65 (78.31%)	136 (77.27%)	0.9596
Infiltrated	17 (23.61%)	18 (21.69%)	40 (22.73%)	

**Table 17:** Analysis of resection margins according to the risk degree of cSCC.

Resection Margins	Very High Risk (VHR)	High Risk (VHR)	Low Risk (LR)	*p*
Free	35 (59.32%)	52 (78.79%)	169 (81.64%)	0.0014
Infiltrated	24 (40.68%)	14 (21.21%)	38 (18.36%)	

**Table 18:** Analysis of tumour-infiltrated versus non-infiltrated resection margins according to the macroscopic appearance of the specimen.

Specimen Shape	Elliptical	Irregular	Nodular	Round-Oval	Triangular	*p*
Total	198 (59.64%)	107 (32.23%)	6 (1.81%)	19 (5.72%)	2 (0.60%)	N/A *
Resection margins	** 165 (83.33%)	** 71 (66.36%)	** 3 (50.00%)	** 15 (78.95%)	** 2 (100.00%)	0.0041
	*** 33 (16.67%)	*** 36 (33.64%)	*** 3 (50.00%)	*** 4 (21.05%)	*** 0 (0.00%)	

* N/A—not assessed, ** free margins, *** tumour-infiltrated margins.

**Table 19:** Macroscopic tumour appearance in relation to inflammatory infiltrate and risk degree.

Tumour Shape	Irregular	Nodular	Round-Oval	*p*
Total	198 (64.71%)	87 (28.43%)	21(8.86%)	N/A *
Inflammatory infiltrate	** 127 (64.80%)	** 55 (63.22%)	** 14 (66.67%)	0.9910
	*** 69 (35.20%)	*** 32 (36.78%)	*** 7 (33.33%)	
LR	120 (60.91%)	60 (30.46%)	17 (8.63%)	0.4407
HR	40 (72.73%)	13 (23.64%)	2 (3.64%)	
VHR	36 (69.23%)	14 (26.92%)	2 (3.85%)	

* NA—not assessed, ** inflammatory infiltrate present, *** absence of inflammatory infiltrate, LR—low risk, HR—high risk, VHR—very high risk.

**Table 20:** Analysis of excised specimens and tumour volumes according to demographic, histopathological, and temporal variables.

	Excisional (Specimen) Analysis					Tumoral Analysis				
	n	Q1 (mm^3^)	Median (mm^3^)	Q3 (mm^3^)	*p*	n	Q1 (mm^3^)	Median (mm^3^)	Q3 (mm^3^)	*p*
Total	331	560	1725	4928	NA	299	55	200	850	N/A *
Female	138	438.3	1492	5745	0.5221	127	49	189	1104	0.8747
Male	193	645	1782	4722		172	60.75	202	813.8	
Rural	108	671	1556	3945	0.7665	97	61.5	200	721.5	0.3181
Urban	164	541.5	1764	4706		156	40.5	142	711	
VHR	59	720	2880	11,200	0.0004	51	70	255	1020	0.0070
HR	66	260.5	717.5	3411		53	37.5	90	251.5	
LR	206	630	1766	4530		195	56	216	975	
Recurrence	18	926	2738	10,388	0.1371	7	18	60	120	0.0360
Non-recurrence	313	543	1680	4797		312	54	200	888.8	
Ulcerated	167	840	2520	7020	<0.0001	160	75	268	1103	0.0003
Non-ulcerated	164	394.8	1075	2690		139	36	108	420	
Free margins	255	585	1728	4620	0.9275	240	54.25	195	813.8	0.8850
Infiltrated margins	76	461.5	1590	7155		59	60	200	990	
Pre-COVID-19	72	390	1945	8700	0.2167	57	72	352	1225	<0.0001
COVID-19	84	587.3	2073	6668		74	137.5	357	2273	
Post-COVID-19	175	560	1484	3900		168	42.75	120	430.5	

* N/A—not assessed; LR—low risk; HR—high risk; VHR—very high risk.

**Table 21:** Correlation of excision specimen and tumour volume with age and year of diagnosis.

Correlation	*p*	*r*	CI (95%)
Excisional (specimen) volume vs. age	0.0001	(+)0.21	(+)0.10–(+)0.31
Excisional (specimen) volume vs. year of diagnosis	0.298	(−)0.058	(−)0.17–(+)0.053
Tumoral volume vs. age	0.0001	(+)0.22	(+)0.11–(+)0.33
Tumoral volume vs. year of diagnosis	<0.0001	(−)0.23	(−)0.3372–(−)0.12
Year of diagnosis vs. age	0.0190	(+)0.13	(+)0.018–(+)0.24

## Data Availability

The data presented in this study are available on request from the first author and corresponding author due to ethical reasons.

## Abbreviations

The following abbreviations are used in this manuscript:

AK	Actinic keratosis
BCC	Basal cell carcinoma
BD	Bowen’s disease
BMI-1	B-cell specific Moloney murine leukemia virus integration site 1
CDH-1	Cadherin-1
CDKN2A	Cyclin-dependent kinase inhibitor 2A
COVID-19	Coronavirus disease 2019
cSCC	Cutaneous squamous cell carcinoma
ΔNp63	Delta-N isoform of tumour protein p63
DNA	Deoxyribonucleic acid
EZH2	Enhancer of zester homologue 2
HPV	Human papillomavirus
HR	High risk
IHC	Immunohistochemistry
IL-1/6/8	Interelukin-1, interelukin-6, interelukin-8
KMTC2C/D	Lysine methyltransferase 2C and 2D
LR	Low risk
MTBCC	Metatypical carcinoma
NCCN	National Comprehensive Cancer Network
NMSC	Non-Melanoma skin cancer
N-RAS	Neuroblastoma RAS Viral oncogene homologue
NOTCH-1	Neurogenic locus notch homologue protein 1
NOTCH-2	Neurogenic locus notch homologue protein 2
p14ARF	Alternate reading frame protein of CDKN2A
p16INK4a	Cyclin-dependent kinase inhibitor 2A isoform p16
p300	E1A binding protein P300
p40	ΔNp63 isoform squamous marker
p63	Tumour protein 63
p53	Tumour protein 53
Rb	Retinoblastoma protein
RIPK4	Receptor interacting serine/threonine protein kinase 4
S100	S100 calcium-binding protein family
SOX10	SRY-Box transcription factor 10
TERT	Telomerase reverse transcriptase
TP53	Tumour protein 53
US	United States
UV	Ultraviolet
VEGF	Vascular endothelial growth factor
VHR	Very high risk
WHO	World Health Organisation
